# Phosphorus Availability Promotes Bacterial DOC-Mineralization, but Not Cumulative CO_2_-Production

**DOI:** 10.3389/fmicb.2020.569879

**Published:** 2020-09-24

**Authors:** Lina Allesson, Tom Andersen, Peter Dörsch, Alexander Eiler, Jing Wei, Dag O. Hessen

**Affiliations:** ^1^Department of Biosciences and Centre for Biogeochemistry in the Anthropocene, University of Oslo, Oslo, Norway; ^2^Faculty of Environmental Sciences and Natural Resource Management, Norwegian University of Life Sciences, Ås, Norway

**Keywords:** dissolved organic carbon-mineralization, lake metabolism, response curves, phosphorus addition, stoichiometry

## Abstract

The current trend of increasing input of terrestrially derived dissolved organic carbon (DOC) to boreal freshwater systems is causing increased levels of carbon dioxide (CO_2_) supersaturation and degassing. Phosphorus (P) is often the most limiting nutrient for bacterial growth and would thus be expected to increase overall mineralization rates and CO_2_ production. However, high carbon (C) to P ratios of terrestrially derived DOC could also cause elevated cell-specific respiration of the excess C in heterotrophic bacteria. Using data from a survey of 75 Scandinavian lakes along an ecosystem gradient of DOC, we estimated *in situ* CO_2_ production rates. These rates showed a unimodal response with DOC-specific CO_2_ production negatively related to DOC:total phosphorus (TP) ratio, and a turning point at 5 mg C L^−1^, indicating higher DOC turnover rates in productive than in unproductive lakes. To further assess the dependency of bacterial respiration (BR) on DOC and P, we monitored CO_2_ production in incubations of water with a gradient of DOC crossed with two levels of inorganic P. Finally, we crossed DOC and P with a temperature gradient to test the temperature dependency of respiration rates [as oxygen (O_2_) consumption]. While total CO_2_ production seemed to be unaffected by P additions, respiration rates, and growth yields, as estimated by ribosomal gene copy numbers, suggest increased bacterial growth and decreased cell-specific respiration under non-limited P conditions. Respiration rates showed a sigmoid response to increasing DOC availability reaching a plateau at about 20 mg C L^−1^ of initial DOC concentrations. In addition to these P and DOC level effects, respiration rates responded in a non-monotonic fashion to temperature with an increase in respiration rates by a factor of 2.6 (±0.2) from 15 to 25°C and a decrease above 30°C. The combined results from the survey and experiments highlight DOC as the major determinant of CO_2_ production in boreal lakes, with P and temperature as significant modulators of respiration kinetics.

## Introduction

Heterotrophic bacteria play a key role in aquatic ecosystems, consuming dissolved organic carbon (DOC) and converting it to carbon dioxide (CO_2_) through bacterial respiration (BR; Del Giorgio et al., 1997; Duarte and Prairie, 2005) and biomass through bacterial production (BP; Del Giorgio and Cole, 1998; Jansson et al., 2006; Berggren et al., 2010).

Bacterial respiration is probably the largest biotic sink of organic carbon on Earth and DOC constitutes a major part of the bulk organic carbon globally (Del Giorgio and Williams, 2005; Drake et al., 2018). Together this makes aquatic bacteria an essential part of the global carbon (C) budget.

The DOC sustaining heterotrophic bacterial metabolism in aquatic ecosystems originates either from primary production within the system (autochthonous DOC) or from terrestrial primary production in the catchment (allochthonous DOC). There is a current trend of increasing transport of terrestrial DOC, to some extent also of total phosphorus (TP) and total nitrogen (TN), to inland waters, caused by factors such as recovery from acidification, climate change, and land use change (Monteith et al., 2007; Kellerman et al., 2015; Finstad et al., 2016; Kritzberg, 2017; Škerlep et al., 2020).

Allochthonous DOC contains a mixture of substances with a variety of molecular size, age, and bioavailability (Neff and Asner, 2001). A large portion of the allochthonous DOC is composed of humic substances, containing aromatic hydrocarbons of high C to nutrient ratios (Mcknight and Aiken, 1998). In humic-rich, low-productive lakes, typical for the boreal zone terrestrially derived substrates often make up the main source of energy and nutrients for bacterial maintenance and growth (Hessen et al., 1990; Karlsson et al., 2007). This decouples the microbial metabolism from the conventional “microbial loop” fueled by autochthonous DOC from algal exudates. In such DOC-rich systems, BR and BP are positively correlated to concentrations of allochthonous DOC rather than to primary production (Jones, 1992; Jansson et al., 2000; Karlsson et al., 2007).

The aromaticity of humic substances causes efficient light absorption and renders DOC prone to photochemical degradation. Allochthonous DOC thus attenuates light and reduces CO_2_ uptake through primary production, while promoting CO_2_ production through both biological and photochemical mineralization. Allochthonous DOC is a key driver of the in-lake partial pressure of CO_2_ (Sobek et al., 2003; Humborg et al., 2010; Larsen et al., 2011). High DOC input renders most boreal lakes net heterotrophic, serving as major conduits of CO_2_ to the atmosphere (Hessen et al., 1990; Cole et al., 1994; Sobek et al., 2003).

The share of the total assimilated organic carbon used for BP is given by the bacterial growth efficiency [BGE = BP/(BP + BR)], determining to what degree bacterial metabolism results in bacterial biomass production or in mineralization of organic carbon (Del Giorgio and Cole, 1998). In planktonic communities, BGE varies substantially and has been shown to depend on the quality rather than the quantity of the DOC (Vallino et al., 1996).

Bacterial carbon utilization efficiency is governed by the nutrient to C ratio of the substrate and availability of inorganic nutrients. Bacteria have a high nutrient demand, such that heterotrophic bacteria may dispose of “excess C” under high C-to-nutrient regimes (Hessen, 1992; Hessen and Anderson, 2008). While bacterial metabolism is often limited by C, N, and P, bacterial biomass accumulation is primarily limited by P and N as these are essential building blocks for RNAs and proteins (Sterner and Elser, 2002). However, there is a trade-off in microbial response to substrate C:P ratios. High C:P promotes increased cell-specific respiration, while elevated P support increased growth and biomass accumulation, thus increasing community respiration while the cell-specific respiration still may be reduced and BGE high (Hessen, 1992). Bacterial degradation of DOC at nutrient sufficiency will most likely result in C allocation to bacterial growth, while nutrient limitation may result in higher respiratory rates as the bacteria dispose of excess C (Smith and Prairie, 2004; Hessen and Anderson, 2008; Berggren et al., 2010).

The C to nutrient ratio thus has great implications for the BGE and the cycling and fate of C in a planktonic habitat. Inorganic P is the most frequently reported limiting nutrient for BP (Vadstein, 2000) and it is mainly the P availability that regulates the use of DOC for growth. Experiments of adding DOC and inorganic P to oligotrophic lake waters have shown that low BGE’s accompanying increased C:P ratios do not necessarily mean that BP is decreasing but rather that BR is increasing (Jansson et al., 2006).

Together with increased run-off and biomass production, air and water temperatures are increasing with the ongoing climate warming (Schneider and Hook, 2010; O’Reilly et al., 2015). Temperature has a fundamental role in regulating the activity and growth of microorganisms (Farrell and Rose, 1967; Madigan et al., 1997). While it can be broadly stated that metabolism increases with temperature up to a certain level, the rate of the exponential increase differs between organisms, reactions, and temperature ranges. Although the rates of both BP and BR increase with temperature, several studies have reported that the temperature dependency of BR is stronger than that of BP and as a consequence, BGE has often been shown to decrease at increasing temperatures (Rivkin and Legendre, 2001; Apple et al., 2006; Berggren et al., 2010; Kritzberg et al., 2010).

Furthermore, the temperature dependency of bacterioplankton metabolic rates interacts with the substrate quantity and quality. Metabolic rates have been shown to be less temperature dependent for heterotrophic bacteria growing on labile autochthonous DOC than when growing on complex and recalcitrant allochthonous DOC (Ylla et al., 2012; Jane and Rose, 2018). In aquatic systems with a DOC pool heavily influenced by terrestrial inputs, increased temperatures are expected to further enhance BR, expanding the role of heterotrophic bacteria as CO_2_ conduits to the atmosphere.

Microbial mineralization of DOC thus depends on several interacting factors. Although we can expect that increased loadings of terrestrially derived DOC and nutrients and enhanced temperatures increase bacterial growth and metabolism, more studies are needed to elucidate how the different environmental factors interact.

In this study, we used chemical and physical data from 75 Scandinavian lakes to estimate in-lake CO_2_ production. The lakes spanned close to orthogonal ecosystem gradients in DOC and TP, allowing us to assess the interactive effects of these two parameters on CO_2_ production. To test for dynamic responses of bacterioplankton respiration to allochthonous DOC concentrations, nutrient availability, and temperature, we additionally performed experimental incubations. During 1-week incubations, we monitored respiration in two experimental set-ups, one addressing CO_2_ production and the other O_2_ consumption. A gradient of DOC concentrations was achieved by adding natural organic matter (NOM; isolates from a Norwegian humic lake obtained through reverse osmosis) to clear lake water.

## Materials and Methods

### Field Sites

**Figure 1:**
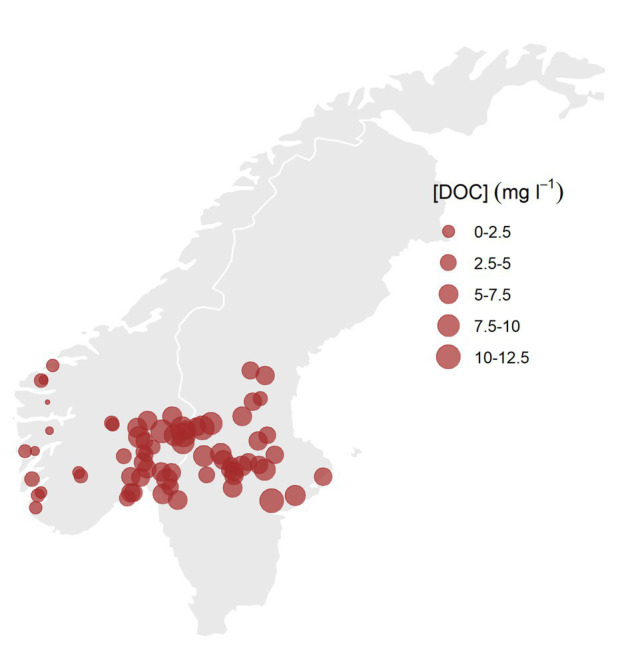
Map of the survey lakes, the size of the circles indicates the concentrations of dissolved organic carbon (DOC; mg L^−1^).

During July and August of 2011, a set of 75 large lakes spread out over a geographical gradient from western Norway to eastern Sweden was sampled (Figure 1). The lakes were chosen to represent wide and close to orthogonal gradients in dissolved organic matter (DOM) and TP. To avoid strong temperature gradients the lakes were chosen within a narrow latitudinal and altitudinal range. All lakes met the following criteria: latitude 57–64°N, altitude <600 m, surface area > 1 km^2^, pH > 5, TP < 30 μg L^−1^, and DOC <30 mg L^−1^. The lakes were sampled by plane in a synoptic survey, and composite samples of a total of 15 L were taken from 0 to 5 m in the central part of each lake during daytime, using an integrating water sampler (Hydro-BIOS, Germany). Vertical temperature profiles and vertical profiles of scalar irradiance (see SI for more detail) were measured using XRX-620 10-channel CTD (RBR Ltd., Canada). Vertical temperature profiles indicated that the thermocline was deeper than 5 m in all lakes. The integrated 0–5 m samples could thus be considered representative of the mixed layers of the lakes.

### Laboratory Analyses of Lake Samples

Concentrations of TP, total organic carbon (TOC), and TN were measured in two accredited laboratories, at the Norwegian Institute for Water Research (NIVA) and at the University of Oslo (UiO). Total inorganic carbon (TIC) was measured at UiO (see SI and Thrane et al., 2014 for details).

Dissolved CO_2_ and O_2_ were measured as headspace concentrations in acid (0.2% HgCl) fixed samples by gas chromatography (GC) analysis (see SI and Yang et al., 2015 for details). Chlorophyll *a* (chl *a*) concentration was measured in two different ways, both by high performance liquid chromatography (HPLC; Schagerl and Donabuam, 2003) and by fluorescence spectrometry after extraction in 96% ethanol. The averages of the two methods (which generally matched well) were used in further analyses. Chromophoric dissolved organic matter (CDOM) optical density [ODCDOM(λ)] of 20 ml filtered lake water (Acrodisc 0.2 μm polyethersulfone membrane syringe filter, Pall Life Sciences) was measured from 400 to 750 nm in steps of 1 nm. According to Mitchell et al. (2002), from which we calculated the absorption coefficient spectra of CDOM [aCDOM(λ); m^−1^].

Area-specific primary production (PP_A_; mg C m^−2^ d^−1^) was calculated using a bio-optical model based on lake-specific phytoplankton absorption coefficients, daily *in situ* irradiance, and the light dependent quantum yield of photosystem II measured by a Pulse Amplitude Modulated (PAM) fluorometer (AquaPen-C 100, PSI Czech Republic; for details see SI and Thrane et al., 2014). As PP_A_ here is a measure of CO_2_ consumption, we can note it as the CO_2_ flux from water to primary producers F_PP_.

Water-air flux of CO_2_ (F_net_) represents the net degassing of CO_2_ from the surface and was calculated from surface CO_2_ concentrations of each lake using Fick’s law of diffusion.

$$F_{net}=k_{CO2}\Delta_{CO2}$$ (1)

where the CO_2_ gas exchange (kCO_2_) coefficient was obtained according to Jähne et al. (1987) and Cole and Caraco (1998; for details see SI and Yang et al., 2015).

### Total CO_2_ Production

From the dataset, it was not possible to distinguish between lateral input of CO_2_ (F_lat_; surface and ground water inflow) and in-lake production of CO_2_ (F_min_; microbial and photochemical mineralization of DOC). The sum of in-lake DOC mineralization and lateral input was therefore used as an estimate of total CO_2_ production (F_tot_ = F_lat_ + F_min_). Assuming a steady state of the CO_2_ saturation deficit, the mass balance due to production, lateral input, consumption, and evasion can be written as:

$$F_{net}=F_{tot}-F_{PP}$$ (2)

And therefore

$$F_{tot}=F_{\min}+F_{lat}=F_{net}+F_{PP}\;$$ (3)

### Experimental Design

To investigate the effects of DOC and P additions on bacterioplankton respiration in more detail, we incubated water samples with a gradient in dissolved NOM concentrations crossing it with two levels of inorganic P. Samples were incubated in the dark using two different experimental set-ups and methods. During the incubations, we monitored bacterioplankton respiration through measurements of either CO_2_ production or O_2_ consumption, depending on the set-up. Samples for microbial biomass were taken upon termination of the experiments with the aim of assessing biomass from a flow-cytometer equipped with a plate reader set-up. However, despite trying with different stains and protocols, the samples were obscured by background scatter from the added DOC, and hence did not provide reliable counts. Ideally, the dynamic responses in CO_2_ and O_2_ should have been verified not only with bacterial counts but also assessment of community response by genetic screening and transcriptomics. This does, however, require a different set-up with larger volumes and a more frequent sampling regime that does not compromise the semi-continuous gas analysis. With the current set-up, we prioritized the gas analysis as the ultimate response output but assessed final microbial biomass from quantitative PCR (qPCR) on filtered samples when terminating the experiment.

## Source of Dom

The DOM gradient was obtained using a NOM isolates from the DOC-rich boreal bog/lake Hellerudmyra close to Oslo, produced within the NOM-typing and the NOMiNiC projects (see Gjessing et al., 1999 and Vogt et al., 2001 for isolation protocol and characterization). This is a powder material up-concentrated from freshwater through reverse osmosis and isolated by freeze-drying of the concentrate. While also containing non-humic material, the major fraction of the NOM isolate consists of humic substances. The carbon fraction of the isolate is 33.7%. The NOM powder was mixed in deionized water to a stock solution of DOM with a DOC concentration of 1,000 mg C L^−1^ and filter sterilized through a 0.2 μm pore size Supor membrane filter (Gelman, CO, USA). While the isolation and up-concentration of the material enriches various elements, the DOM retains its “natural” properties (Gjessing et al., 1999; Vogt et al., 2001), and should be superior to artificial sources of DOC.

### Preparation of the Media

The DOM stock solution was diluted to the desired DOC concentrations in sterile filtered (0.2 μm pore size Supor membrane filter; Gelman, CO, United States) drinking water from the tap. This water comes from the oligotrophic and pristine lake Maridalsvannet in the municipality of Oslo, Norway. The water is treated following protocols for drinking water. The processing includes alkalization/carbonazation by marble and CO_2_, coagulation and particle separation in Actiflo followed by two media filter, and UV irradiation for disinfection. Besides disinfecting, the UV treatment also lowers the CDOM content in the water. The treatments do not eliminate essential trace metals and macronutrients in the water, giving background concentrations of DOC and total N of 2 mg L^−1^ and 0.01 mg L^−1^, respectively, while the background total P was below detection limits.

The samples were inoculated with 1% of the total sample volume of fresh water from a stream draining Hellerudmyra from where the DOM was isolated. The inoculum was filtered (2 μm) to remove large particles and protists.

### Experimental Set-Up 1: CO_2_ Production

We used 14 levels of DOC additions between 0 and 50 mg L^−1^ (0 mg L^−1^, 2.5 mg L^−1^, 5 mg L^−1^, 7.5 mg L^−1^, 10 mg L^−1^, 12.5 mg L^−1^, 15 mg L^−1^, 17.5 mg L^−1^, 20 mg L^−1^, 22.5 mg L^−1^, 25 mg L^−1^, 34 mg L^−1^, 41 mg L^−1^, and 50 mg L^−1^). The DOC gradient was crossed with two levels of PO_4_-P additions (0 and 2 μmol L^−1^). To make sure that N was not limiting, 30 μmol L^−1^ each of NO_3_-N and NH_4_-N, resulting in a total of 60 μmol L^−1^ N, were added to all samples. In samples with P additions, the C:N:P ratio thus ranged between 82:30:1 and 2160:30:1.

The incubations were carried out in the dark in a temperature-controlled water bath at 20°C. Samples of 50 ml were transferred into 122 ml, acid washed glass vials equipped with acid washed magnetic stirrers. The vials were crimp sealed with butyl rubber septa. Before incubation, the vials were washed with HeO_2_ (80/20%) by 6 cycles of evacuation and filling using a manifold, while stirring the samples at 400 rpm to remove CO_2_ and nitrogen gas (N_2_). The samples were incubated constantly stirred (400 rpm) for 10 days at 20°C, while CO_2_ production and O_2_ uptake were measured automatically by GC every 6 h using the robotized set-up described by Molstad et al. (2016) with some modifications. Since these vials contained 50 ml water and 70 ml air, the O_2_ uptake was small relative to the large amount of O_2_ in the headspace and we could not measure uptake rates with sufficient precision.

### Experimental Set-Up 1: Ribosomal RNA Gene Copy Numbers

To approximate bacterial growth yields we measured bacterial 16S ribosomal RNA (rRNA) gene copy numbers using a qPCR protocol (Savio et al., 2015). In short, 40 ml of water from each incubation were filtered through 0.2 μm Supor PES membrane filters (Pall Corporation, CA, United States) at the end of the experiment. Filters were stored at −80°C until DNA extraction was performed using the Dneasy PowerSoil kit as recommended by the manufacturer (Qiagen, Germany). Total DNA concentration was assessed using a Qubit ds DNA Broad-Range Assay (London, United Kingdom), and 16S rRNA genes were quantified using a bacteria-specific qPCR. qPCR reactions contained 2.5 μl DNA extract as the template and 0.2 μM each of the primers 8F and 338 (Frank et al., 2007; Fierer et al., 2008) targeting the V1-V2 region of most bacterial 16S rRNA genes and iQ SYBR Green Supermix (Bio-Rad Laboratories, Hercules, USA). Samples were run in triplicates together with a dilution series of the ZymoBIOMICS Gut Microbiome Standard (Zymo Research, Irvine, USA) to obtain 16S rRNA gene copy numbers in each incubation.

### Experimental Set-Up 2: Dissolved Oxygen Consumption

The second experimental set-up was designed with fewer DOC levels (0 mg L^−1^, 25 mg L^−1^, and 50 mg L^−1^) plus an additional crossing with four temperatures (10, 15, 25, and 30°C). All treatments were run in quadruplicates.

The incubations were carried out in the dark, placed in a water bath in a climate chamber to assure stable temperature. Dissolved oxygen concentrations were measured over 7 days of incubation with a SensorDish Reader (SDR; resolution: ±0.4% O_2_ at 20.9% O_2_, PreSens GmbH, Regensburg, Germany) using non-invasive fluorescence sensor spots placed in the bottoms of 5 ml vials, which were measured by optodes. The vials were washed with 70% ethanol and baked at 80°C for 10 h before filling them with sample and leaving them in the incubator for temperature equilibration for 2 h. After the equilibration, we inoculated the samples and filled the vials to the top, leaving no headspace. The vials were then closed and additionally sealed with parafilm to avoid gas diffusion. Vials with deionized water were used as controls to check for O_2_ leakage. Dissolved oxygen concentrations were recorded automatically every 15 s. O_2_ consumption rates were may represent CO_2_ production rates, assuming a respiratory quotient (RQ: mole CO_2_ produced/mole O_2_ consumed) equal to 1. Although humic-rich substances are completely oxidized at an RQ of 0.9 (Dilly, 2001), anabolic processes contributes to higher RQs than catabolic respiration alone (Dilly, 2003; Berggren et al., 2012). We therefore believe that an RQ of 1 is an appropriate assumption. However, in the analysis, we use the rates of O_2_ consumption that indirectly represent CO_2_ production rates (see below), and thus the RQ value does not affect the results. In some treatments, the O_2_ was depleted toward anoxia, but this should not affect our analysis since the initial slopes of the uptake curves, when the incubations still were oxic, to estimate bacterial growth.

Temperature sensitivity of the respiration rates were analyzed using the Q_10_ coefficient as the relative change in rate when increasing the temperature by 10°C.

$$Q_{10}={\left(\frac{R_{2}}{R_{1}}\right)}^{\left(\frac{10}{T_{2}-T_{1}}\right)}$$ (4)

where R is the rate, here the respiration rate (μmol CO_2_ L^−1^ h^−1^) and T is the temperature in centigrade.

### Modeling the Respiration Curves

Respiration curves and growth rates were modeled using the packages mgcv (Wood, 2011) and gratia (Simpson, 2018) in R (R Core Team, 2020). A generalized additive model (gam) with simple factor smoothers on time and grouped by experimental unit was fitted to all the measured time-series data. The fitted curves were then differentiated, using the derivatives function from the gratia package (Simpson, 2018) to estimate the time course of the net rate of change in each experimental unit. From the fitted derivatives we calculated maximum O_2_ consumption or CO_2_ production rates for each experimental unit. The time until the maximum rate was reached was used as a measure of the lag phase.

### Statistics

All data analysis was performed using the open-source software R version 4.0.2 (R Core Team, 2020). Lake variables were checked for normality and log-transformed where needed. Correlations are reported using Pearson’s correlation coefficients and all error estimates are given in standard errors. For the statistical modeling, we used the mgcv package (Wood, 2011) fitting gam models for prediction of the dependent variables. To test the dependency of total CO_2_ production (F_tot_; mg C m^−2^ d^−1^), we used a gam model with smoothers on each of the explanatory variables DOC (mg L^−1^), TP (μg L^−1^), TN (mg L^−1^), TIC (mg L^−1^), SUVA_400_ (L mg-C^−1^ m^−1^), and temperature (°C). Predictive variable selection was done by applying additional shrinkage on the null space of the penalty with the select = TRUE argument in the mgcv::gam function, as recommended by Marra and Wood (2011). The resulting model has all smoothers that are not necessary for the fit as close to zero as possible.

For the analysis of the experimental results, individual gam models were fitted to samples with and without P additions (and the three levels of DOC concentrations in the second experiment), respectively. In the case of experimental set-up 1, gams were fitted with DOC concentration as the explanatory variable, while in experimental set-up 2 temperature was used. We also performed an analysis of covariance to test for treatment effects of P (experimental set-up 1) or P and temperature (experimental set-up 2).

## Results

### Lake Survey

Concentrations of both DOC and TP varied largely among lakes, with DOC concentrations ranging between 0.25 and 12.9 mg L^−1^ and TP between 0.5 and 27.5 μg L^−1^. Although lakes had been selected for orthogonality between DOC and TP, there was a correlation between the two variables (*p* < 0.001, *r* = 0.61, log-log). Still a considerable scatter indicates reasonable orthogonality. Other important cross-correlations that need to be considered in data interpretation are positive relationships of TN with both TP and DOC concentrations (Supplementary Figure S1).

Dissolved CO_2_ concentrations spanned two orders of magnitude (0.82–133 μmol L^−1^) with the majority of the lakes being strongly supersaturated with CO_2_ as indicated by an average CO_2_ of more than twice that at atmospheric equilibrium. This supersaturation conveys evasion of CO_2_ to the atmosphere from most lakes and net-heterotrophy of the lake systems during the sampling campaign. As expected, the level of CO_2_ saturation was positively correlated with DOC concentration (*p* < 0.001; *r* = 0.52) and negatively correlated with O_2_ saturation (*p* < 0.001; *r* = −0.69; Supplementary Figure S2) and thus reflects the role of DOC as a driver of heterotrophy. There was no correlation between TIC and CO_2_ saturation deficit, suggesting that in-lake processes were the main cause of CO_2_ supersaturation.

**Figure 2:**
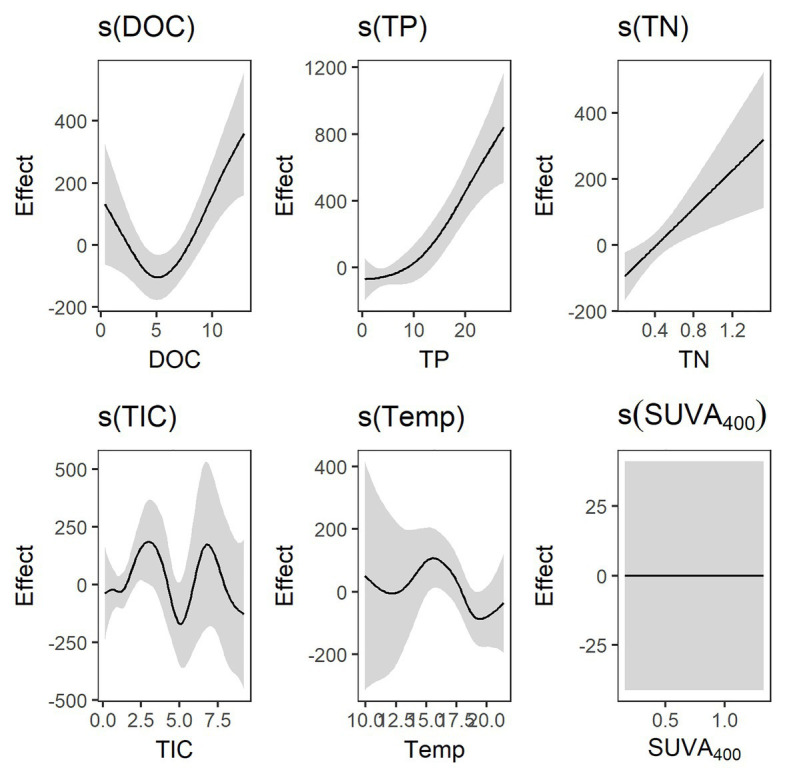
Result plot of the generalized additive models (*gams*) predicting total carbon dioxide (CO_2_) production (F_tot_; mg C m^−2^ d^−1^). The effect of DOC (mg L^−1^) was strong and clearly unimodal with a minimum around 5 mg L^−1^. Total phosphorus (TP; μg L^−1^) and total nitrogen (TN; mg L^−1^) had strong linear effects. The effects of total inorganic carbon (TIC; mg L^−1^) and temperature (°C) were weak, while SUVA_400_ (L mg-C^−1^ m^−1^) had no effect on total CO_2_ production.

Areal primary production (PP_A_) was not significantly correlated to CO_2_ concentrations, providing no support for primary production being boosted by CO_2_ in CO_2_ rich lakes. Still, PP_A_ was weakly but negatively related to DOC concentrations (*p* = 0.05; *r* = −0.23, log-log; Thrane et al., 2014), and somewhat (but non-significant) lower in high CO_2_ lakes than in lakes with low CO_2_ concentrations. In addition, PP_A_ was negatively related to the DOC: TP ratio (*p* < 0.001; *r* = −0.60; Supplementary Figure S3A). Total CO_2_ (F_tot_) production here represents both CO_2_ produced in lakes and CO_2_ coming into lakes from the surroundings *via* ground water and run-off and was estimated as the sum of net CO_2_ evasion and a real primary production (F_tot_ = F_min_ + F_lat_ = F_net_ + F_PP_). The gam model explained 77% of the total deviance in total CO_2_ production with strong effects of DOC, TP, and TN. TIC and temperature had weak effects and SUVA had no effect on total CO_2_ production (Figure 2). Relating total CO_2_ production to DOC concentration revealed a unimodal response to increased DOC concentrations with a minimum value at around 5 mg L^−1^ (Figure 2). Carbon concentration-specific CO_2_ production [F_tot_: (mg C m^−2^ d^−1^)/DOC(mg C m^−2^)] was positively related to PP_A_ (*p* < 0.001, *r* = 0.60, log-log) and was thus, similar to PP_A_, decreasing with an increasing DOC:TP ratio (Supplementary Figure S3B).

### Incubation Experiments

To test for dynamic responses underlying the patterns observed across lakes, two experimental incubations were performed, one addressing the production of CO_2_, and the other the consumption of O_2_. Both incubations were performed in the dark, hence they reflect heterotrophic microbial mineralization.

In the CO_2_ production experiment, DOC concentration had a positive effect on CO_2_ production in all samples, regardless of P level. However, P had a strong effect on the kinetics of CO_2_ production and hence the shape of the CO_2_ accumulation curves (Supplementary Figure S4). While all treatments showed non-linear CO_2_ accumulation similar to logistic growth, the exponential phase was more distinct and steeper in the treatments receiving P additions. CO_2_ production was faster and leveled off earlier and more distinctively in P-spiked than in P-limited samples. This effect was most pronounced for initial DOC concentrations up to 25 mg L^−1^. The lag phase was more pronounced in P-spiked samples but decreased in length with increasing DOC concentrations. At DOC concentrations >25 mg L^−1^, CO_2_ concentrations kept increasing at a slower pace after an initial exponential phase without reaching a plateau (Supplementary Figure S4).

**Figure 3:**
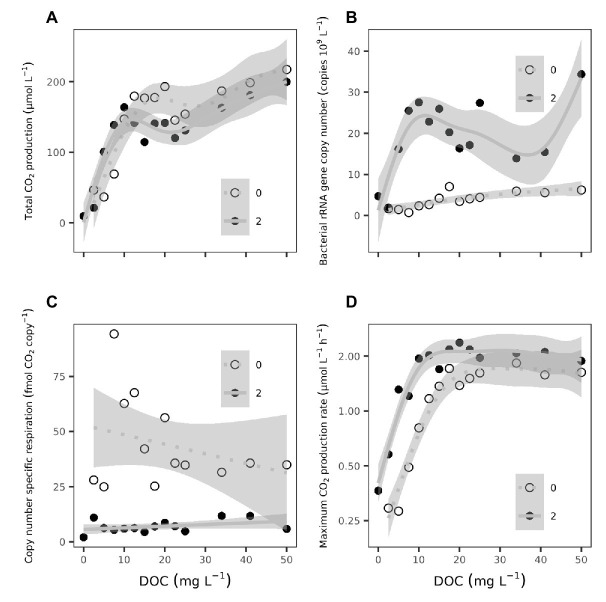
The response of **(A)** total CO_2_ production (μmol L^−1^) during the entire incubation (240 h), **(B)** yield in bacterial 16S ribosomal RNA (rRNA) gene copy number (copies L^−1^), **(C)** bacterial 16S rRNA gene copy number-specific respiration (fmol CO_2_ copy^−1^), and **(D)** maximum CO_2_ production rates (μmol L^−1^ h^−1^) to increased DOC concentrations (mg L^−1^). The solid and dotted lines are fitted *gams* to samples with and without phosphorus (P) additions, respectively, with shaded areas representing the confidence interval of the *gams*. Statistics of the *gams* can be found in Supplementary Table S1.

The total amount of CO_2_ produced during the laboratory incubation increased monotonously with initial DOC concentration until reaching a threshold value (~10 mg L^−1^), above which cumulative CO_2_ production appeared to be independent of the amount of DOC supplied, before increasing again with DOC concentration above 30 mg L^−1^, albeit at lower rate (Figure 3A). Treatments with P additions had a somewhat lower threshold value than treatments without P additions. Still, the total amount of CO_2_ produced during the incubations were similar in samples with or without P additions (Supplementary Table S1), while higher rRNA gene copy numbers were observed in P-spiked samples (Figure 3B; Supplementary Table S1). Consequently, estimates of rRNA gene copy number-specific respiration, used here as a proxy for bacterial growth yield, were lower in P-spiked than non-spiked samples (Figure 3C; Supplementary Table S1), indicating less respiration per unit biomass produced and hence larger growth yields due to P addition. There was also an increase in rRNA gene copy numbers with increasing DOC concentrations in P-spiked and non-spiked samples (Figure 3B), while estimates of rRNA gene copy number-specific respiration showed no clear trends with regards to DOC concentrations (Figure 3C).

Similar to total CO_2_ production, the maximum observed respiration rates at any time during the incubation, increased steadily with increasing DOC up to 20 mg L^−1^ (Figure 3D). Below this threshold value, maximum inducible respiration rates were clearly higher in P spiked than in P limited samples. Maximum inducible respiration rates increased by about 9% for each mg DOC L^−1^ up to 20 mg L^−1^, regardless of P level. At DOC concentrations >20 mg L^−1^, the increase in respiration rates with increasing DOC halted abruptly and stayed constant with similar rates at the two P levels.

The fraction of the DOC pool that was respired to CO_2_ during the course of the experiments was similar across the P levels, but decreased with DOC concentration. At concentrations above the threshold of 20 mg L^−1^, the respired fraction of the DOC pool was about 5% (Supplementary Figure S6).

**Figure 4:**
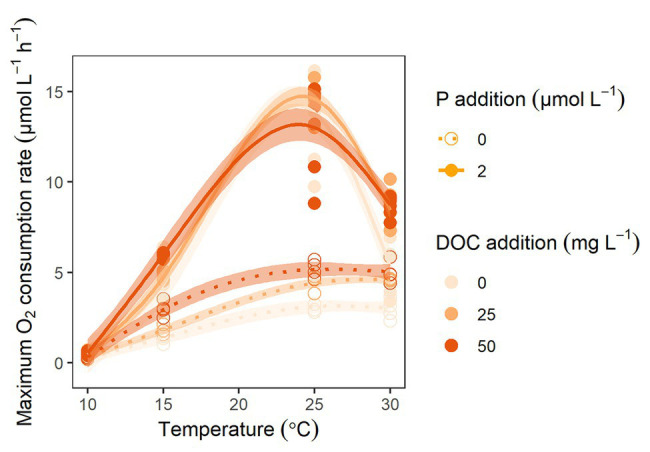
The response of maximum oxygen (O_2_; μmol L^−1^ h^−1^) consumption rates to increased temperatures. The solid and dotted lines are fitted gam curves [y ~ s(x)] curves to samples with and without P additions, respectively. The color gradient represents DOC additions, going from light for no additions to dark for 50 mg C L^−1^.

In the O_2_ consumption experiment, the dynamic response in O_2_ uptake to DOC and P as well as temperature was tested. Similar to the CO_2_ production curves, the shapes of the O_2_ consumption curves differed substantially depending on P level with a more pronounced exponential phase in samples receiving P additions than in samples without P additions (Supplementary Figure S5). Respiration rate was also highly dependent on temperature (Figure 4). Maximum O_2_ consumption rates were mainly related to P level and temperature (Supplementary Table S1), while the regression estimate of the DOC concentration effect was non-significant (Supplementary Table S1).

Respiration rates in the 10°C incubation were close to zero, possibly reflecting that respiration had not started to increase before the incubation was stopped and that the incubation time thus was too short with the lowest temperature. Respiration rates increased from 15 to 25°C with a Q_10_ of 2.6 (±0.2) and no significant difference in Q_10_ between samples of different P levels. From 25 to 30°C, the respiration rates decreased with a Q_10_ < 1. Here the Q_10_ also differed between P spiked (0.44 ± 0.09) and P limited (0.94 ± 0.05) samples.

## Discussion

### DOC and P Availability Regulating Total CO_2_ Production in Lakes

In this study, we analyzed data from a lake survey comprising 75 Scandinavian lakes chosen to represent gradients in DOM and P concentrations. Chemical and physical data from the lakes were used to estimate total CO_2_ production. As it was not possible to distinguish between in-lake production and lateral input of CO_2_ (from inflowing rivers or groundwater input), total CO_2_ production was used to lump both sources. Notwithstanding, we found a strong relationship between O_2_ and CO_2_ saturation deficits (*r* = −0.70; Supplementary Figure S2). The intercept was not significantly different from zero and lakes that were saturated with O_2_ were thus also saturated with CO_2_, indicating that microbial respiration was the predominant source of CO_2_ in the lakes. Furthermore, we found no correlation between TIC and CO_2_ deficit, suggesting that processes within the lakes rather than lateral input regulate the CO_2_ supersaturation. Although some studies have shown that DIC input from the catchment plays a larger role for explaining CO_2_ evasion from lakes than previously thought (Maberly et al., 2013; Leith et al., 2015; Weyhenmeyer et al., 2015), the largest contributor to CO_2_ supersaturation in the studied lakes was most probably microbial mineralization.

The observed unimodal response of total CO_2_ production to increased DOC concentrations (Figure 2), however, suggests a shift in substrate from mainly autochthonous to predominantly allochthonous DOC. Autochthonous DOC is generally more bioavailable and of higher nutritious value with a lower C:P ratio (Søndergaard et al., 1995). When DOC of both phytoplankton and terrestrial origin is available, heterotrophic bacteria prefer the former as substrate for catabolic processes (Kritzberg et al., 2004). Estimated CO_2_ production rates decreased with increasing DOC concentration until a minimum was reached at around 5 mg L^−1^. Correspondingly, primary production rates are commonly reported to increase with DOC concentrations until around 5 mg C L^−1^, after which the rates are declining (Karlsson et al., 2007; Seekell et al., 2015; Tanentzap et al., 2017). This unimodal response is likely reflecting a trade-off between nutrients associated with DOC and the increasing light attenuation caused by CDOM. Modest increases in DOC may also be beneficial by blocking out short-wave UV-radiation (Palen et al., 2002). Above 5 mg L^−1^, an increasing portion of the DOC pool is of terrestrial origin and CO_2_ production rates thus increase linearly with increasing DOC concentrations.

The DOC concentration-specific CO_2_ production, i.e., the rates of CO_2_ production per unit of DOC concentration, was positively related to PP_A_, indicating that a larger share of the DOC pool was respired in more productive lakes. The DOC:TP ratio had a negative effect on PP_A_, and consequently, the DOC:TP ratio also had a negative effect on the DOC concentration-specific CO_2_ production (Supplementary Figure S3). This may seem to contradict the notion that BR increases with increased C:P ratios (Jansson et al., 2006). However, on a community level low BGE at high C:P ratios has been coupled to decreasing BP rates rather than increasing BR rates (Smith and Prairie, 2004). Higher DOC concentration-specific CO_2_ production indicates faster DOC turnover in the low than in the high C:P lakes. A larger share of the DOC pool is degraded, probably accompanied with higher bacterial density in productive than in unproductive lakes. This implies a more bioavailable DOC pool in productive than in unproductive lakes, and could also suggest that this is explained by a lower C:P ratio of the substrate.

### Experimental Validation of Drivers

While lake gradients may provide general patterns, the mechanistic drivers can only be revealed experimentally. To disentangle the role of DOC relative to P, we conducted two experiments. First, we measured BR along a gradient of DOC concentrations crossed with two levels of inorganic P concentrations. Since this DOC was an isolate from a humic lake (see section “Materials and Methods”), it represented primarily allochthonous C. We found clear differences in the kinetics of CO_2_ production between P spiked and P-limited samples. The CO_2_ accumulation curves of P-spiked samples showed a pronounced exponential phase until reaching a plateau, similar to a bacterial growth curve reaching substrate limitation (Supplementary Figure S4). The kinetic patterns suggested that P-spiking boosted respiratory rates leading to substrate limitation earlier during incubation. The longer lag phase in P-spiked samples could be explained by a major shift in community composition. P-limited samples would represent a situation with minor shifts in community composition. The rRNA gene copy number-specific respiration suggests that cell-specific respiration increased under P-limited conditions. Accordingly, Smith and Prairie (2004) found cell-specific respiration to be negatively related to P supply. They further report that on a per cell basis, BR explained the greatest amount of variation in BGE. This would suggest a higher BGE in P-spiked than in P-limited samples in our experiment, provided a higher BR cell^−1^ in P-limited compared to P-spiked samples, as reflected by the lower rRNA gene copy number-specific respiration in P-spiked samples. More P, however, also releases the bacteria from P-limitation, hence causing higher metabolic activity. The balance between respiration due to excess C (i.e., under high substrate C:P) or respiration powered by increased metabolic activity under elevated P (i.e., low C:P) is not straight forward, since increased P also would stimulate bacterial growth and thus community metabolism. This unpredictability is corroborated by our results as rRNA gene copy number-specific respiration in non-spiked samples showed a negative trend with increasing DOC concentrations, while under P addition a significant increase in rRNA gene copy number-specific respiration could be observed. Previous studies with C and P manipulations, showed good correspondence between bacterial biomass and CO_2_ production (Hessen et al., 1994), but in the absence of reliable day-to-day microbial counts we cannot fully resolve this stoichiometric response at the cellular versus the community level.

The cumulative amount of CO_2_ produced during the incubation was higher in P-spiked than in P-limited samples at low initial DOC concentrations (<10 mg L^−1^; Figure 3A). In the intermediate DOC range (12.5–25 mg L^−1^), however, this trend shifted and more of the available DOC was respired in P-limited than in P-spiked samples (Supplementary Figure S4). The larger cumulative CO_2_ production in P-limited samples above ~20 mg L^−1^ initial DOC could reflect that the slow-growing population assimilated the substrate at a slower pace and that the major share of the assimilated DOC was used for maintenance and hence was respired. However, this is not corroborated by rRNA gene copy number-specific respiration. The bioavailable fraction of DOC was consumed more rapidly when P was available than when P was limiting, reflecting faster DOC turnover similar to the findings in the lake survey.

We added a fixed amount of naturally isolated DOC at the beginning of the experiment, and microbial assimilation and respiration depleted a large share of the bioavailable fraction of this DOC during the course of the experiment. In a natural environment, however, fresh DOC would enter the aquatic system, e.g., by rainfall and influx from the catchment *via* rivers, brooks, or surface run-off, eventually by vertical mixing, constantly refreshing the DOC pool. Further, recalcitrant DOC would undergo photochemical processing that breaks down high molecular weight humic acids into more bioavailable substrates (Bertilsson and Tranvik, 1998). Under such conditions, mineralization would continue to increase with increasing DOC concentrations, and continuous supply of P would support higher rates of mineralization. This is in accordance with the findings from the lake survey where total CO_2_ production rates increased with decreasing DOC:TP ratios (Supplementary Figure S3B) and increasing TP.

In the laboratory experiments, respiration rates increased with increasing DOC concentration up to 20 mg L^−1^. This increase in respiration with DOC was in accordance with the increase in total CO_2_ production rates at increased DOC concentration >5 mg L^−1^ found in the lake survey (Figure 2). Many boreal lakes have DOC concentrations below 20 mg L^−1^ (in our lake survey, for instance, the maximum value was 12.9 mg C L^−1^) and a continued increase in BR with increased terrestrially derived DOC up to about 20 mg L^−1^ could be expected.

The respired fraction of DOC in the laboratory experiment was small (max 18%) and decreased with increased DOC concentration beyond a distinct peak at 12.5 mg DOC L^−1^ (Supplementary Figure S6). Since the source of DOC and thus the bioavailable share was the same in all samples, this suggests a lower mineralization efficiency with increased concentrations of DOC. In high DOC (>25 mg L^−1^) treatments, the CO_2_ accumulation curves of neither P-spiked nor P-limited samples reached a plateau (Supplementary Figure S4), suggesting that mineralization would continue beyond the 200 h of incubation, although at lower pace.

In the experiment using O_2_-consumption as proxy for microbial activity (Supplementary Figure S5), temperature and P were strong predictors of O_2_ while DOC was a poor predictor. A temperature increase from 15 to 25°C yielded an increase in respiration rates by a factor of 2.6 (±0.2) with no significant difference between P levels. This Q_10_ value is in accordance with reported Q_10_ values for physiological processes (Pomeroy and Wiebe, 2001). While respiration rates did not increase further at temperatures >25°C in P-limited samples, they decreased in P-spiked samples. Between 25 and 30°C the Q_10_ thus differed significantly between P spiked (0.44 ± 0.09) and P limited (0.94 ± 0.05) samples. This difference in Q_10_ may reflect a difference in bacterial taxonomic composition between treatments of different P levels with a faster growing and less robust community in P spiked than in P limited samples. Although there is a trend of increasing water temperatures (O’Reilly et al., 2015), an increase in temperature >25°C is unlikely to occur within the nearest future. Around 15–25°C may therefore be the most relevant temperature range for natural systems.

The observed temperature response together with the temperature sensitivity of secondary production being higher than that of primary production (Brown et al., 2004; Apple et al., 2006), further reinforce the idea that net heterotrophy in lakes will increase with increasing temperatures, which also would lead to increased emissions of CO_2_ (Sobek et al., 2003). While we found no temperature effect on neither lake CO_2_ production, nor CO_2_ flux in the lake survey, likely reflecting that temperature measurements were snapshots, we further speculate that the narrow temperature gradient sampled in our study is overridden by a dynamic natural environment with several potentially confounding and fluctuating factors. Moreover, the role of primary production as a regulator of CO_2_ is minor in these lakes. Given the positive temperature effect on microbial metabolism (Farrell and Rose, 1967), the ongoing rise in temperature, along with current browning (O’Reilly et al., 2015; Solomon et al., 2015), means that an increased CO_2_ output from boreal lakes and rivers can be expected. The availability of P will serve as an additional regulator of carbon emissions, with increased respiration rates and DOC turnover rates in DOC-rich lakes where the role of primary production is small, and vice versa in cases where P is declining (Thrane et al., 2014).

Bacterioplankton respiration is a key process for converting organic carbon to CO_2_ in aquatic ecosystems (Williams and Del Giorgio, 2005). This mineralization of DOC driven by microbial respiration is accompanied by O_2_ consumption, often with an assumed RQ of 1, yet this quotient depends on substrate properties and metabolic states (Dilly, 2003; Berggren et al., 2012; Allesson et al., 2016). Similar to the CO_2_ production rates in experimental set-up 1 and the higher O_2_ consumption rates in P-spiked samples in experimental set-up 2 could represent a situation with higher BGE than in P-limited samples. As anabolic processes often are accompanied with elevated RQ’s, we can expect some differences in RQ between samples of different P levels and possibly some underestimation of respiration rates in P-spiked samples by the use of an RQ value of 1. However, such differences in RQ values between P levels would make the differences in respiration rates between treatments more pronounced and the conclusions thus are still valid. High rates of heterotrophic respiration together with low rates of primary production promote oxygen depletion with major consequences for aquatic life as well as redox processes and biogeochemical cycling of C, N, P, and other elements. While increased respiration rates following increased DOC concentrations may favor primary production due to increased access to CO_2_ as well as nutrients associated with DOC, increased browning and thereby increased light attenuation would most probably result in a net decline in primary production and thus increased net heterotrophy (Thrane et al., 2014; Seekell et al., 2015), with a tentative turning point around 5 mg C L^−1^.

In conclusion, the DOC concentration regulates the overall respiratory output of CO_2_ (and consumption of O_2_), while additions of P changes the dynamics by boosting respiration, as did elevated temperatures. The overall respiratory outcome depends on substrate stoichiometry and the potentially different cell-specific responses versus community responses; i.e., larger biomass will generate a larger total CO_2_ output despite lower cell-specific respiration. The dynamic responses revealed in the small-scale batch experiments do not necessarily capture inter-lake responses to changing DOC:TP ratios, partly because “fresh” DOC becomes available for microbial respiration due to inflow and mixing *in situ*, and partly because phytoplankton responses will impact the net CO_2_ balance. Also the full nature of biotic uptake and recycling can clearly not be captured, but the combination of a gradient lake surveys together with the laboratory experiments revealed DOC as the major determinant of CO_2_ production in boreal lakes, with P as a significant modulator.

## Data Availability Statement

The raw data supporting the conclusions of this article will be made available by the authors, without undue reservation.

## Author Contributions

LA and DH conceived the idea. LA, DH, PD, and AE conducted the experiments. All authors were involved in the analysis of data and final writing. All authors contributed to the article and approved the submitted version.

### Conflict of Interest

The authors declare that the research was conducted in the absence of any commercial or financial relationships that could be construed as a potential conflict of interest.
