# Corrigendum: Phosphorus Availability Promotes Bacterial DOC-Mineralization, but Not Cumulative CO_2_-Production

**DOI:** 10.3389/fmicb.2020.614974

**Published:** 2020-11-23

**Authors:** Lina Allesson, Tom Andersen, Peter Dörsch, Alexander Eiler, Jing Wei, Dag O. Hessen

**Affiliations:** ^1^Department of Biosciences and Centre for Biogeochemistry in the Anthropocene, University of Oslo, Oslo, Norway; ^2^Faculty of Environmental Sciences and Natural Resource Management, Norwegian University of Life Sciences, Ås, Norway

**Keywords:** dissolved organic carbon-mineralization, lake metabolism, response curves, phosphorus addition, stoichiometry

In the original article, there was a mistake in Figure 2 as published. The wrong figure was published. The figure caption remain unvaried. The corrected Figure 2 appears below.

**Figure 2:**
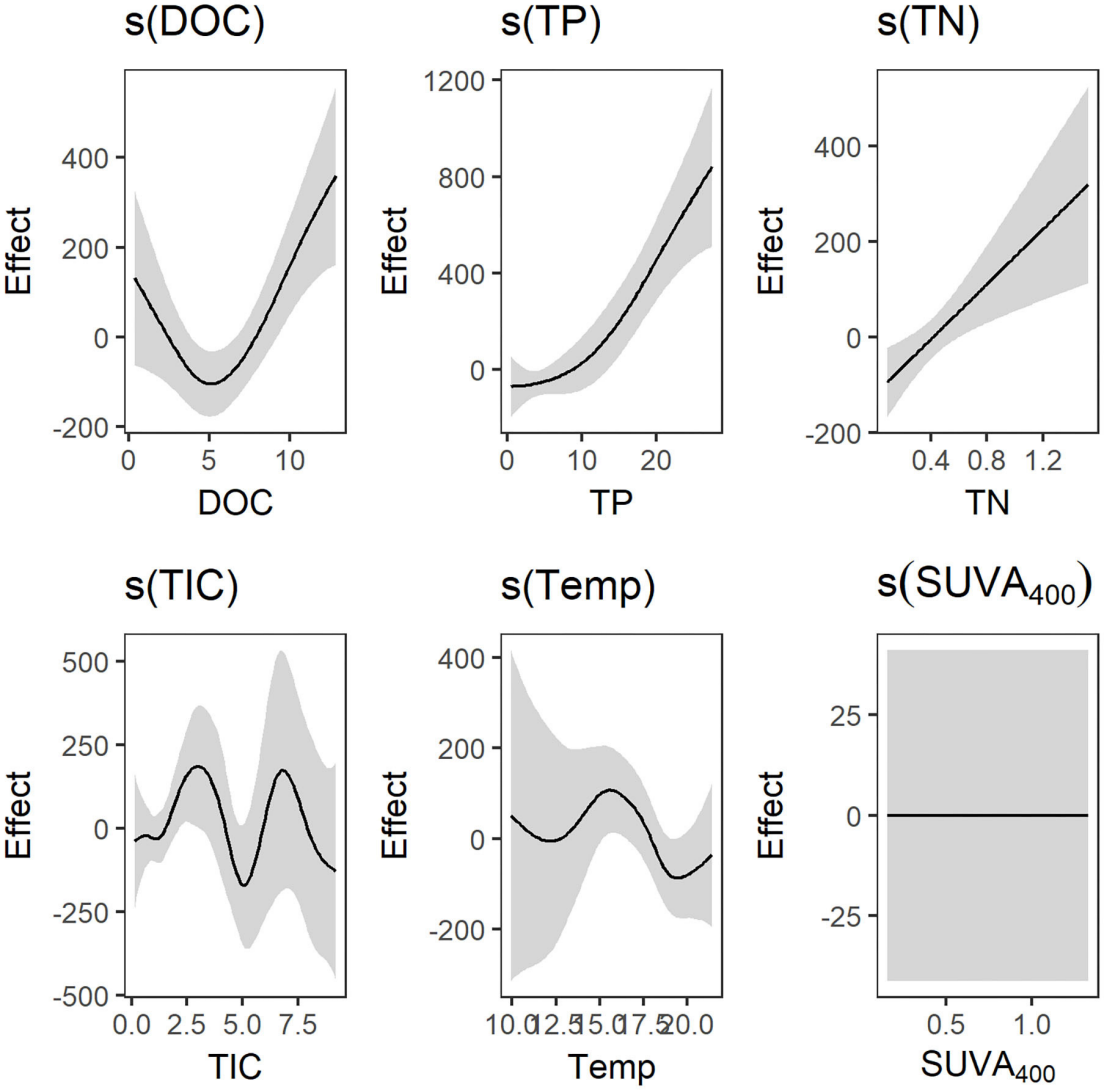
Result plot of the generalized additive models (*gams*) predicting total carbon dioxide (CO_2_) production (F_tot_; mg C m^−2^ d^−1^). The effect of DOC (mg L^−1^) was strong and clearly unimodal with a minimum around 5 mg L^−1^. Total phosphorus (TP; μg L^−1^) and total nitrogen (TN; mg L^−1^) had strong linear effects. The effects of total inorganic carbon (TIC; mg L^−1^) and temperature (°C) were weak, while SUVA_400_ (L mg-C^−1^ m^−1^) had no effect on total CO_2_ production.

The authors apologize for this error and state that this does not change the scientific conclusions of the article in any way. The original article has been updated.

