# Supplementary material for: Phosphorus Availability Promotes Bacterial DOC-Mineralization, but Not Cumulative CO2-Production
Source: Front Microbiol. 2020 Sep 24;11:569879. doi: 10.3389/fmicb.2020.569879 (PMC7541949; doi:10.3389/fmicb.2020.569879)
Supplement: Supplementary file 1 [file Data_Sheet_1.docx]

Supporting information materials and methods

Vertical profiles of scalar irradiance in the photosynthetically active radiation (PAR) region (400 -700 nm; E_0_) were measured using a spherical irradiance sensor (BioSpherical instruments) attached to a 10 channel CTD profiler (WRW620. RBR Ltd., Canada). The sensor was lowered at a rate of approximately 20 cm s^-1^ with a sampling rate of 6 Hz. In order to correct for temporal changes in irradiance caused by for example wave action and clouds during the CTD cast, we regressed the log-transformed E_0_ against depth (z) for each ten sampling point (i.e. sliding windows). The vertical attenuation coefficient for scalar PAR (K_0_PAR) was then estimated by taking the median of the distribution of these slopes.

Measurements of concentrations of total phosphorus (TP), total organic carbon (TOC) and total nitrogen (TN) were carried out both at the Norwegian Institute for Water Research (NIVA) and at the University of Oslo (UiO). Regressions between the measurements at the two laboratories showed no systematic differences (TP: *R*^2^ = 0.77, residual standard error (*RSE*) = 2.27 µg l^-1^; TOC: *R*^2^ = 0.99, *RSE* = 0.25 mg l^-1^; TN: *R*^2^ = 0.91, *RSE* = 81 µg l^-1^) and the averages of the results were used in the subsequent analysis. DOC was calculated as the difference between the total organic carbon (TOC) and particulate organic carbon (POC). TOC was measured by infrared CO_2_ detection after catalytic high temperature combustion (Shimadzu TOC-VWP analyser (UiO), or Phoenix 8000 TOC-TC analyser (NIVA)).
POC was measured on an elemental analyser (Flash EA 1112 NC, Thermo Fisher Scientific, Waltham, Massachusetts, USA) through rapid combustion in pure oxygen of particulates captured on a pre-combusted GF/C-filter. The major part (> 95 %) of the TOC was in dissolved form (DOC). At UiO, TOC was measured together with total inorganic carbon (TIC). TP was measured on an auto-analyser as phosphate after wet oxidation with peroxodisulfate. The two labs measured TN in different ways. UiO measured TN on unfiltered samples by detecting nitrogen monoxide by chemiluminescence using a TNM-1 unit attached to the Shimadzu TOC-VWP analyser, and NIVA measured TN through detection of nitrate after wet oxidation with peroxodisulfate in a segmented flow auto-analyser.
For gas analyses, water from the composite water sample (integrated from 0 to 5 m depth) was gently let into 120 ml glass serum vials without bubbling. The samples were fixed with 0.2% HgCl and sealed with gas-tight butyl rubber stoppers (see Yang et al. (2015) for details). Prior to analysis, the vials were stored dark and cold (4 ºC). In order to prepare a 20-30 ml headspace, a gentle helium pressure (needle valve) was applied to the top of the bottle volume replacing ca. 40 ml of sample with pure He, before venting the bottles to 1 atmosphere. Equilibration between liquid and headspace was achieved by shaking the bottles horizontally at 150 rpm for 2 h at room temperature. Headspace concentrations of CO_2_ and O_2_ were determined by automated gas chromatography (GC; Model 7890A, Agilent, CA, USA).

In brief, the bio-optical model calculating area specific primary production (PP_A_) is based on estimating the in vivo rate of light absorption by phytoplankton, and subsequently electron transport rates (ETRs) through photosystem II (PSII) using information about the light-dependent quantum yield of the PSII photochemistry. ETR can further be converted to a rate of gross carbon fixation by assuming an appropriate value for the quantum yield of CO_2_ fixation (Kromkamp and Forster, 2003, Suggett et al., 2010). While the method could be sensitive to phytoplankton community composition, it has gained increased interest over the last two decades because it offers a fast and inexpensive way of obtaining PP_A_ estimates (see Thrane et al (2014) for details). A comparison of this method and empirical estimates for PP_A_ in boreal lakes demonstrated good accordance (Thrane et al., 2014). The method is thus a feasible tool for assessment of primary production across a large number of sites. It also avoids many of the pitfalls of ^14^C-bottle incubation, which in any case could not have been applied in this kind of synoptic survey.

CO_2_ flux

We used Fick’s law of diffusion to calculate the water-air flux of CO_2_ (F_net_; mmol m^-2^ d^-1^) from lake surface CO_2_ concentrations.:

$F_{net}=k_{CO2}\Delta_{CO2}$ (1)

where k_CO2_ (m d^-1^) is the CO_2_ gas exchange coefficient at a given temperature and ∆_CO2_ (mmol m^-3^) is the CO_2_ deficit from concentrations at equilibrium with the atmosphere, obtained using Henry’s law. k_CO2_ was estimated for each lake using the gas transfer velocity (m d^-1^) for a gas-temperature combination with a Schmidt number of 600 (k_600_; CO_2_ at 20 °C) according to Jähne et al. (1987):

$k_{CO2}=k_{600}\left( \frac{{Sc}_{CO2}}{600} \right)^{-x}$ (2)

where x = 2/3 if wind speed ≤ 3ms^-1^ and x = 0.5 if wind speed > 3m s^-1^, Sc is the temperature dependent Schmidt number for CO_2_. k_600_ is estimated from the wind speed according to Cole and Caraco (1998):

$k_{600}=2.07+{0.215 U}_{10}^{1.7}$ (3)

Hourly wind speed data at 10 m above ground (U_10_ in equation 9) at all 75 lakes were extracted from the Norwegian Reanalysis Archive (NORA10) and aggregated into July-August means.

COLE, J. J. & CARACO, N. F. 1998. Atmospheric exchange of carbon dioxide in a low-wind oligotrophic lake measured by the addition of SF6. *Limnology and Oceanography,* 43**,** 647-656.

JÄHNE, B., MÜNNICH, K. O., BÖSINGER, R., DUTZI, A., HUBER, W. & LIBNER, P. 1987. On the parameters influencing air-water gas exchange. *Journal of Geophysical Research: Oceans,* 92**,** 1937-1949.

KROMKAMP, J. C. & FORSTER, R. M. 2003. The use of variable fluorescence measurements in aquatic ecosystems: differences between multiple and single turnover measuring protocols and suggested terminology. *European Journal of Phycology,* 38**,** 103-112.

SUGGETT, D. J., PRÁŠIL, O. & BOROWITZKA, M. A. 2010. *Chlorophyll a fluorescence in aquatic sciences: methods and applications*, Springer.

THRANE, J.-E., HESSEN, D. O. & ANDERSEN, T. 2014. The Absorption of Light in Lakes: Negative Impact of Dissolved Organic Carbon on Primary Productivity. *Ecosystems,* 17**,** 1040-1052.

YANG, H., ANDERSEN, T., DÖRSCH, P., TOMINAGA, K., THRANE, J.-E. & HESSEN, D. O. 2015. Greenhouse gas metabolism in Nordic boreal lakes. *Biogeochemistry,* 126**,** 211-225.
