# Supplementary material for: Phosphorus Availability Promotes Bacterial DOC-Mineralization, but Not Cumulative CO2-Production
Source: Front Microbiol. 2020 Sep 24;11:569879. doi: 10.3389/fmicb.2020.569879 (PMC7541949; doi:10.3389/fmicb.2020.569879)
Supplement: Supplementary file 2 [file Data_Sheet_2.docx]

Figure S1. Correlation matrix between dissolved organic carbon (DOC; mg L^-1^), total phosphorus (TP; µg L^-1^), and total nitrogen (TN; mg L^-1^). All variables are log-transformed.

Figure S2. Correlation matrix between DOC (mg L^-1^), CO_2_ deviance from saturaion (%), O_2_ deviance from saturation (%), and concentration of chlorophyll *a* (chl *a*; µg L^-1^).

Figure S3. The response of a) area specific primary production (PP_A_; mg C m^-2^ d^-1^), and b) DOC specific CO_2_ production rates (F_tot_ (mg C m^-2^ d^-1^)/DOC (mg C m^-2^)) to increased DOC:TP ratio.

Figure S4. Cumulative CO_2_ production over incubation time. The figure is divided into boxes of DOC additions starting from top left with no additions to bottom right with 50 mg C L^-1^. Dotted and solid lines represent treatments without and with P additions, respectively.

Figure S5. Cumaltive O_2_ consumption over incubation time. The figure is divided into boxes of DOC (top row: no addition; middle row: 25 mg C L^-1^; bottom 50 mg C L^-1^), and temperature (from 10 ºC in the left column to 30 ºC in the right column). The colors represent treatments without (red) and with (blue) P additions, respectively.

Figure S6. The respired fraction of the added DOC versus DOC concentration (mg L^-1^) open circles are treatments without P and filled circles are treatments with 2 µmol L^-1^ P addition.
