# Supplementary material for: Phosphorus Availability Promotes Bacterial DOC-Mineralization, but Not Cumulative CO2-Production
Source: Front Microbiol. 2020 Sep 24;11:569879. doi: 10.3389/fmicb.2020.569879 (PMC7541949; doi:10.3389/fmicb.2020.569879)
Supplement: Supplementary file 3 [file Data_Sheet_3.docx]

| **Response variable** | **Factor** | **edf^*^** | **F-value** | **R^2^_adj_** | **Dev. Expl.^#^** | **Effect^§^** |
| --- | --- | --- | --- | --- | --- | --- |
| **Experimental set-up 1** |  |  |  |  |  |  |
| total CO_2_ production | s(DOC):P0  s(DOC):P2 | 7.372  7.731 | 23.98  20.27 | 0.935 | 97.5 % | P* |
| 16S rRNA gene copy number | s(DOC):P0  s(DOC):P2 | 1.493  8.931 | 7.273  56.04 | 0.98 | 98.9 % | P*** |
| copy number specific respiration | s(DOC):P0  s(DOC):P2 | 7.548  1.000 | 3.748  0.703 | 0.813 | 88.2 % | P*** |
| maximum CO_2_ production rate | s(DOC):P0  s(DOC):P2 | 2.825  3.689 | 26.46  26.89 | 0.91 | 93.6 % | P*** |
| **Experimental set-up 2** |  |  |  |  |  |  |
| maximum O_2_ consumption rate | s(temp):P0:DOC0  s(temp):P2:DOC0  s(temp):P0:DOC25  s(temp):P2:DOC25  s(temp):P0:DOC50  s(temp):P2:DOC50 | 1.000  1.990  1.000  1.983  1.608  1.982 | 6.49  92.98  15.04  104.18  11.13  89.71 | 0.844 | 84.8% | P***  DOC* |

**Table S1.** Results of a generalized additive model (GAM) from the two experiments using non-parametric smoothers.

^*^ edf is the estimated degree of freedom accounting for the smoothing function.

^#^ Deviance explained by the model with all factors.

^§^ Significance of P-level (experimental set-up 1) and P and DOC levels (experimental set-up 2) on the response variable. Significant codes: *** <0.001; ** <0.01; * <0.05.

| **Variable tested** | **explanatory** | **Estimate** | **t-value** | **p-value** | **R^2^_adj_** | **p-value** |
| --- | --- | --- | --- | --- | --- | --- |
| **Experimental set-up 1** |  |  |  |  |  |  |
| total CO_2_ production | DOC  P | 2.946  -17.90 | 5.650  -1.227 | <0.001  0.232 | 0.553 | <0.001 |
| 16S rRNA gene copy number | DOC  P | 1.93 × 10^8^  1.57 × 10^10^ | 2.305  6.690 | 0.0301  <0.001 | 0.642 | <0.001 |
| copy number specific respiration | DOC  P | -16.1  0.374 | -0.811  -6.730 | 0.425  <0.001 | 0.626 | <0.001 |
| maximum CO_2_ production rates | DOC  P | 0.028  0.539 | 4.724  3.257 | <0.001  <0.005 | 0.531 | <0.001 |
| **Experimental set-up 2** |  |  |  |  |  |  |
| maximum O_2_ consumption rate | DOC  P  Temperature | 0.554  0.365  3.903 | 1.784  11.415  7.270 | 0.077  <0.001  <0.001 | 0.563 | <0.001 |

**Table S2.** Results from analysis of covariance (ANCOVA) using data from the two experiments.
